# Down but not out

**DOI:** 10.7554/eLife.53363

**Published:** 2019-12-23

**Authors:** Erin T Larragoite, Adam M Spivak

**Affiliations:** School of MedicineUniversity of UtahSalt Lake CityUnited States

**Keywords:** HIV, viral latency, latent reservoir, Monkeys, SIV, antiretroviral therapy, Virus

## Abstract

A new study in monkeys suggests that treating HIV infection early with antiretroviral therapy reduces the number of latent viruses, but has little impact on viral reactivation when treatment stops.

**Related research article** Pinkevych M, Fennessey CM, Cromer D, Reid C, Trubey CM, Lifson JD, Keele BF, Davenport MP. 2019. Predictors of SIV recrudescence following antiretroviral treatment interruption. *eLife* **8**:e49022. doi: 10.7554/eLife.49022

When you imagine viruses hiding from your immune system, you may think of the herpes simplex virus, responsible for cold sores and genital herpes, or the varicella-zoster virus, which causes shingles. These viruses can persist for decades in a hibernation-like state known as latency and avoid detection by our immune system (Kennedy et al., 2015). Latent viruses can sometimes awaken and begin replicating once more, causing symptoms and spreading infection (Lieberman, 2016).

The human immunodeficiency virus (HIV) is similarly a master escape artist. It forms a latent reservoir early in infection, allowing the virus to evade the immune system and to survive long-term. People living with HIV take daily medications known as combination antiretroviral therapy (or ART) to prevent the virus from replicating. However, ART is not a cure, as it does not target viruses that are already latent. These drugs are therefore given for the lifetime of an individual: stopping treatment, even for a few weeks, will allow the latent viruses to reactivate and rekindle active infection. People with HIV can expect to have long, healthy lives due to ART, but latent viruses will always be present in their body.

Finding a cure for HIV requires being able to eliminate or control the latent viral reservoir. However, some key questions still need to be addressed to achieve this goal. How, when and where does HIV establish latency? What is the size of the latent reservoir, and the best way to measure it? What triggers latent viruses to reactivate? Now, in eLife, Miles Davenport, Brandon Keele and colleagues from the University of New South Wales and the Frederick National Laboratory for Cancer Research – including Mykola Pinkevych as first author – report when the latent reservoir is established in a non-human primate model of HIV and how much of the reservoir can be reactivated (Pinkevych et al., 2019).

In this study, the team infected rhesus macaques with simian immunodeficiency virus (SIV), a precursor virus which is endemic in African monkeys and genetically similar to HIV (Williams and Burdo, 2009). At some point during the 20^th^ century, SIV jumped from non-human primates into humans; this cross-species transmission event, coupled with rapid evolution, allowed the virus to efficiently spread in people and to create the ongoing HIV pandemic (Keele et al., 2006).

Pinkevych et al. began by infecting rhesus macaques with an engineered SIV containing over 10,000 unique randomized sequences of DNA; once sequenced, these ‘barcodes’ allow individual viruses to be identified (Fennessey et al., 2017). The monkeys were then treated with antiretroviral therapy 4, 10, or 27 days after infection. These intervals simulate acute (4 days), early (10 days), or late intervention (27 days) with ART in humans. The drugs were given for approximately a year, and the virus was completely suppressed in all animals. The treatment was then stopped and the latent virus was allowed to reactivate. Using genetic sequencing and mathematical modeling, the team determined the size of the latent reservoir of SIV and how it would reactivate.

In the monkeys, starting the treatment four days after infection did not block the formation of the latent reservoir, but did reduce its size by approximately 100-fold compared to later ART initiation. Similarly, people who begin ART within days of acquiring HIV have an extremely small reservoir compared to those who start treatment later (Luzuriaga et al., 2015; Henrich et al., 2017). Despite these large differences in overall reservoir size, once ART was stopped the latent viruses reactivated at similar rates in acute, early, and late-treated animals (Figure 1). Pinkevych et al. therefore conclude that the majority of viruses which have the potential to reactivate are establishing latency early after infection.

**Figure 1.**
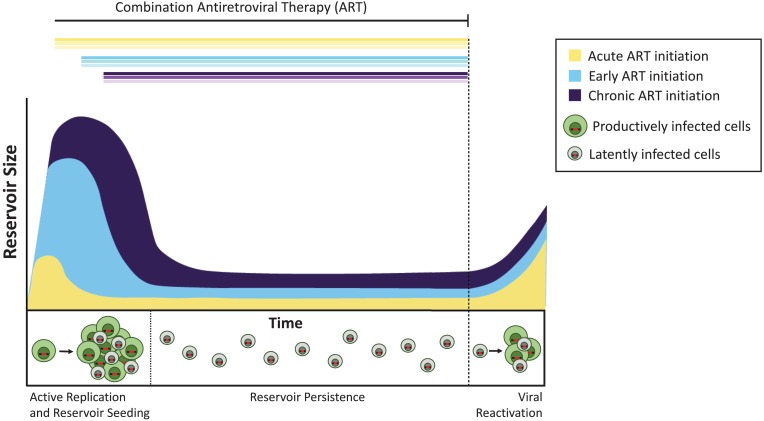
The timing of antiretroviral therapy influences the size of the latent reservoir. Without treatment, cells infected with actively replicating virus (productively infected cells; shown in green) create infectious viruses. A minority of infected cells contain viruses that can persist indefinitely as a latent reservoir (latently infected cells; shown in gray), and these viruses can potentially be reactivated at a later date. Treatment administered during the acute phase of infection (that is, within days or weeks of primary infection; yellow curve) results in a smaller latent reservoir than when treatment is initiated early (within six months of infection, light blue) or during chronic infection (more than 6 months since infection, violet). If treatment is stopped (dashed line), the virus reactivates from these reservoirs at similar levels to rekindle active infection and re-seed the latent reservoir.

To explore why the timing of the treatment did not seem to influence the rate of reactivation, the team measured the level of genetic mutations in the latent viruses. If viruses accumulate more damaging mutations the longer they are actively replicating in the body, this would suggest animals receiving delayed ART could carry a larger proportion of latent viruses that are defective and cannot reawaken. However, the team found that the majority of viruses (more than 80%) were genetically intact across all animals, regardless of when treatment started. This is quite different to what happens with HIV infection in humans, where most of the latent viruses contain major genetic mutations and deletions, leaving just a small fraction (between 2% and 11%) that are capable of reactivation (Ho et al., 2013; Bruner et al., 2019).

A recent study, which evaluated the dynamics of the HIV reservoir in people on stable ART, identified another discrepancy between this SIV model and HIV latency in humans. Despite ongoing ART, latent HIV can sometimes spontaneously reactivate and the viruses become detectable in the blood for a short while. In humans, these viral ‘blips’ are phylogenetically linked to a viral reservoir established not just at initial infection, but across years of untreated infection (Jones et al., 2018). The reasons underlying these differences are not well understood and represent important areas for ongoing research.

Despite differences between non-human primate models and human HIV infection, the work by Pinkevych et al. confirms that viral latency is established extremely early after infection. These results indicate that antiretroviral therapy should be started as soon as possible to control HIV infection and reduce latent reservoir size. Much is still unknown about how HIV latency is established and maintained, especially under treatment; however SIV models will remain an important tool to understand how to eradicate the latent reservoir.

## References

[bib1] Bruner KM, Wang Z, Simonetti FR, Bender AM, Kwon KJ, Sengupta S, Fray EJ, Beg SA, Antar AAR, Jenike KM, Bertagnolli LN, Capoferri AA, Kufera JT, Timmons A, Nobles C, Gregg J, Wada N, Ho YC, Zhang H, Margolick JB, Blankson JN, Deeks SG, Bushman FD, Siliciano JD, Laird GM, Siliciano RF (2019). A quantitative approach for measuring the reservoir of latent HIV-1 proviruses. Nature.

[bib2] Fennessey CM, Pinkevych M, Immonen TT, Reynaldi A, Venturi V, Nadella P, Reid C, Newman L, Lipkey L, Oswald K, Bosche WJ, Trivett MT, Ohlen C, Ott DE, Estes JD, Del Prete GQ, Lifson JD, Davenport MP, Keele BF (2017). Genetically-barcoded SIV facilitates enumeration of rebound variants and estimation of reactivation rates in non-human primates following interruption of suppressive antiretroviral therapy. PLOS Pathogens.

[bib3] Henrich TJ, Hatano H, Bacon O, Hogan LE, Rutishauser R, Hill A, Kearney MF, Anderson EM, Buchbinder SP, Cohen SE, Abdel-Mohsen M, Pohlmeyer CW, Fromentin R, Hoh R, Liu AY, McCune JM, Spindler J, Metcalf-Pate K, Hobbs KS, Thanh C, Gibson EA, Kuritzkes DR, Siliciano RF, Price RW, Richman DD, Chomont N, Siliciano JD, Mellors JW, Yukl SA, Blankson JN, Liegler T, Deeks SG (2017). HIV-1 persistence following extremely early initiation of antiretroviral therapy (ART) during acute HIV-1 infection: an observational study. PLOS Medicine.

[bib4] Ho YC, Shan L, Hosmane NN, Wang J, Laskey SB, Rosenbloom DI, Lai J, Blankson JN, Siliciano JD, Siliciano RF (2013). Replication-competent non-induced proviruses in the latent reservoir increase barrier to HIV-1 cure. Cell.

[bib5] Jones BR, Kinloch NN, Horacsek J, Ganase B, Harris M, Harrigan PR, Jones RB, Brockman MA, Joy JB, Poon AFY, Brumme ZL (2018). Phylogenetic approach to recover integration dates of latent HIV sequences within host. PNAS.

[bib6] Keele BF, Van Heuverswyn F, Li Y, Bailes E, Takehisa J, Santiago ML, Bibollet-Ruche F, Chen Y, Wain LV, Liegeois F, Loul S, Ngole EM, Bienvenue Y, Delaporte E, Brookfield JF, Sharp PM, Shaw GM, Peeters M, Hahn BH (2006). Chimpanzee reservoirs of pandemic and nonpandemic HIV-1. Science.

[bib7] Kennedy PG, Rovnak J, Badani H, Cohrs RJ (2015). A comparison of herpes simplex virus type 1 and varicella-zoster virus latency and reactivation. Journal of General Virology.

[bib8] Lieberman PM (2016). Epigenetics and genetics of viral latency. Cell Host & Microbe.

[bib9] Luzuriaga K, Gay H, Ziemniak C, Sanborn KB, Somasundaran M, Rainwater-Lovett K, Mellors JW, Rosenbloom D, Persaud D (2015). Viremic relapse after HIV-1 remission in a perinatally infected child. New England Journal of Medicine.

[bib10] Pinkevych M, Fennessey CM, Cromer D, Reid C, Trubey CM, Lifson JD, Keele BF, Davenport MP (2019). Predictors of SIV recrudescence following antiretroviral treatment interruption. eLife.

[bib11] Williams KC, Burdo TH (2009). HIV and SIV infection: the role of cellular restriction and immune responses in viral replication and pathogenesis. APMIS.

